# Complete occlusion of right pulmonary artery in Behçet disease

**DOI:** 10.1002/rcr2.594

**Published:** 2020-06-02

**Authors:** Yohei Korogi, Nozomi Tanaka, Hajime Yoshifuji, Junichi Tazaki, Takeshi Kubo, Kiminobu Tanizawa

**Affiliations:** ^1^ Department of Respiratory Medicine, Graduate School of Medicine Kyoto University Kyoto Japan; ^2^ Department of Rheumatology and Clinical Immunology, Graduate School of Medicine Kyoto University Kyoto Japan; ^3^ Department of Cardiovascular Medicine, Graduate School of Medicine Kyoto University Kyoto Japan; ^4^ Department of Diagnostic Imaging and Nuclear Medicine, Graduate School of Medicine Kyoto University Kyoto Japan

**Keywords:** Behçet disease, bronchoalveolar lavage, occlusion, pulmonary artery, vasculo‐Behçet

## Abstract

When a patient with Behçet disease presents with haemoptysis, pulmonary vascular involvement should be considered.

## Graphical abstract

Here, we describe a case of complete occlusion of right pulmonary artery seen in a patient with Behçet disease.

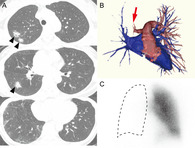

## Clinical Image

**Figure 1:**
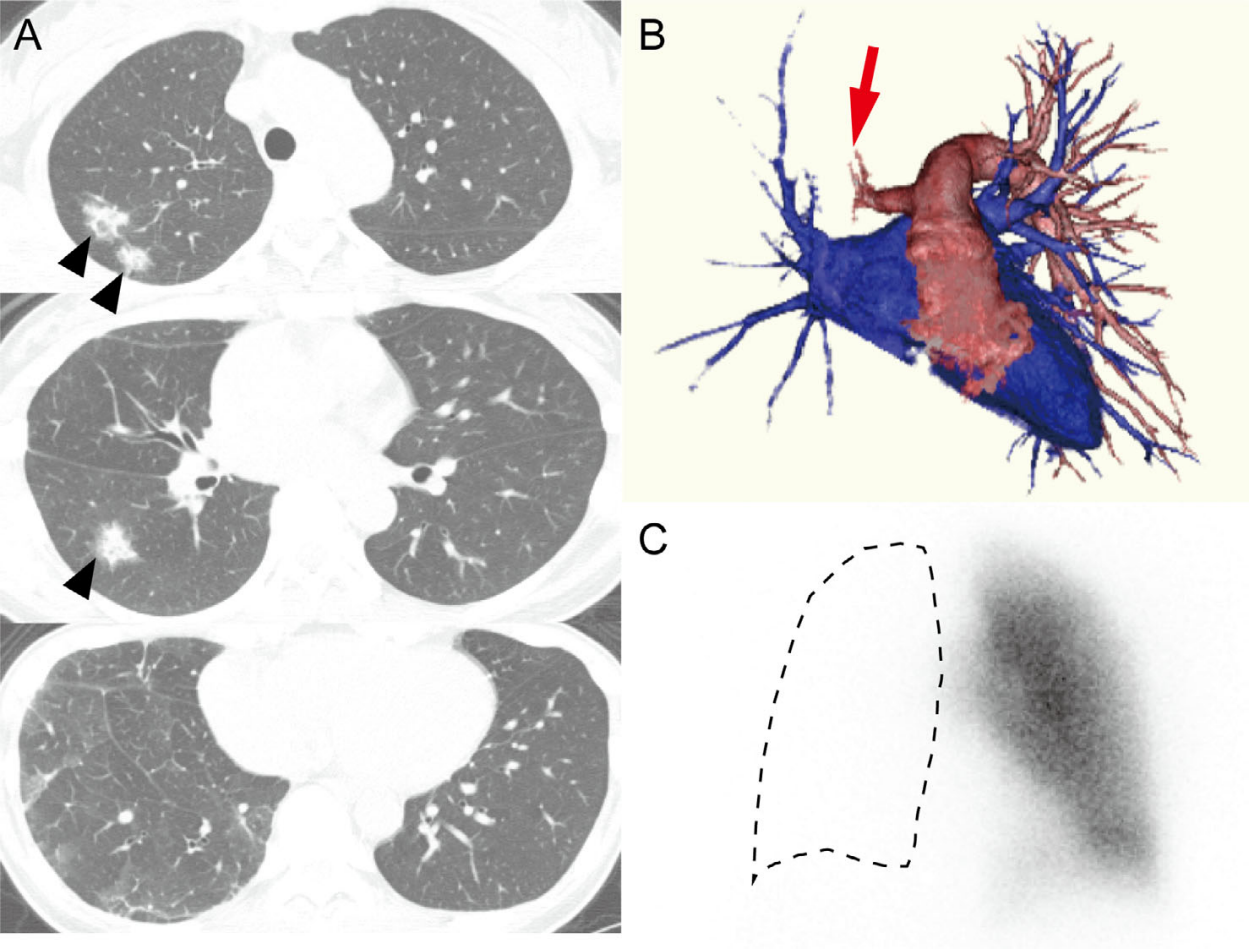
(A) High‐resolution computed tomography (CT) of the chest showed multiple consolidations (arrowheads) and ground‐glass opacities with interlobular septal thickening and decreased pulmonary vessel shadows in the right lung. (B) CT angiography showed complete occlusion of the right pulmonary artery (red arrow). (C) Perfusion scintigraphy revealed a perfusion defect of the right lung (dotted outline).

A 36‐year‐old Japanese female with a seven‐year history of Behçet disease (BD) presented with cough and haemoptysis. High‐resolution computed tomography (CT) showed multiple opacities in the right lung that were refractory to antibiotics (Fig. 1A). Bronchoalveolar lavage fluid (BALF) from the right lower lobe was mildly bloody with haemosiderin‐laden macrophages, suggesting vascular involvement. CT angiography and perfusion scintigraphy revealed complete occlusion of the right pulmonary artery (PA) (Fig. 1B, C). Fluorodeoxyglucose–positron emission tomography–CT showed uptake in the thickened walls of the aorta and its branches. Deep venous thrombosis was not detected with contrast‐enhanced CT and ultrasonography, and serum d‐dimer level was normal. These vascular lesions were diagnosed as the arterial involvement of BD because she had typical oral, genital, and skin manifestations of BD for years. She had HLA‐A*26/A*33 and B*39/B*44, and was negative for B*51 but positive for A*26, both of which are associated with BD. The bloody BALF was considered to be caused by pulmonary infarction. After four weeks of treatment with 45 mg/day of prednisolone, inflammation of the aorta and its branches was improved, whereas the right PA occlusion was unchanged. Vascular involvement was reported in approximately 10% of patients with BD [1], and the aorta and PAs were shown to be affected in 7.6% and 19% of vascular BD patients, respectively [2]. As arterial involvement is associated with a lower long‐term survival rate among BD patients, early detection of such lesions is important in order to start appropriate treatment and improve the long‐term prognosis.

### Disclosure Statement

Appropriate written informed consent was obtained for publication of this case report and accompanying images.
